# Defining the specificity and function of a human neutralizing antibody for Hepatitis B virus

**DOI:** 10.1038/s41541-022-00516-7

**Published:** 2022-10-21

**Authors:** Saket Jhajharia, Fritz Lai, Heng Boon Low, Kiren Purushotorman, Bhuvaneshwari D/O Shunmuganathan, Conrad En Zuo Chan, Rachel Hammond, Hans-Jürgen Netter, Qingfeng Chen, Seng Gee Lim, Paul A. MacAry

**Affiliations:** 1 Department of Medicine, National University of Singapore, Singapore, Singapore; 2 Institute of Molecular and Cell Biology, Agency for Science, Technology, and Research (A*STAR), Singapore, Singapore; 3 Life Sciences Institute, National University of Singapore, Singapore, Singapore; 4 Biological Defence Programme, DSO National Laboratories, Singapore, Singapore; 5 Victorian Infectious Diseases Reference Laboratory (VIDRL), Melbourne Health, The Peter Doherty Institute, Melbourne, VIC Australia; 6 National University Hospital, Division of Gastroenterology and Hepatology, Singapore, Singapore

**Keywords:** Hepatitis B, VDJ recombination

## Abstract

Hepatitis B Virus (HBV) is a hepadnavirus that is the principal pathogen underlying viral liver disease in human populations. In this study, we describe the isolation and characterization of a fully human monoclonal antibody for HBV. This HuMab was isolated by a combinatorial screen of the memory B-cell repertoire from an acute/recovered HBV-infected patient. Lead candidate selection was based upon strong binding and neutralizing activity for live HBV. We provide a detailed biochemical/biophysical, and subclass characterization of its specificity and affinity against all of the principal HBV genotypes combined with a functional analysis of its in vitro activity. We also demonstrate its potential as a prophylaxis/therapy in vivo using human liver chimeric mouse models for HBV infection. These data have important implications for our understanding of natural human immunity to HBV and suggest that this potentially represents a new antibody-based anti-viral candidate for prophylaxis and/or therapy for HBV infection.

## Introduction

Hepatitis B Virus (HBV) infection accounts for 1.34 million deaths each year and WHO has estimated that 296 million individuals are chronically infected worldwide^1^. HBV causes an acute or chronic infection, with a high possibility of the latter resulting in liver cirrhosis and hepatocellular carcinoma (HCC), which are the leading causes of HBV—associated mortality^1,2^. Current therapeutic options for HBV have an efficacy of 1–2% annually in achieving a functional cure^3^. The pivotal importance of an effective B-cell/antibody response for achieving a functional cure was shown in patients receiving B-cell depleting therapies-this results in the reactivation of HBV in resolved individuals^4–6^.

Hepatitis B Immunoglobulins (HBIG) is currently the frontline prophylactic agent employed in at-risk individuals^7,8^. However, HBIG is plagued by limited availability, batch variation, and low specific activity^7,9^. The lack of efficacious therapeutic and prophylactic options translates into a clear requirement for new modalities to improve the clinical management of HBV.

There are three membrane-embedded surface proteins (L, M, and S) on HBV with a common S antigenic region^10^. The S region contains the “a” determinant spanning from residues 99 to 160 and is thought to be the major anti-HBs epitope binding domain^11^. Antibodies targeting the “a” determinant have been reported to be potent neutralizers making them ideal candidates for testing as prophylactic or therapeutic reagents^12^.

Several human or humanized antibodies against HBsAg have been previously isolated against recombinant viral proteins using a variety of discovery methods including: Monoclonal hybridoma technology^13,14^; Phage-FAB display^9,15,16^; Humanized mice^17^; and human B-cell cultures^18,19^. These antibodies have been tested in preclinical models for neutralization and effector functions^20,21^. One previous clinical study using fully human mAbs termed HepeX-B (NCT00228592) was terminated in 2015^22^. Moreover, an antibody candidate termed HB-C7A (GC1102) is currently undergoing phase IIB/2 clinical trials (NCT03801798) in Korea as a replacement for polyclonal HBIG for pre/post-transplantation use. In addition, an antibody termed K1R127 has been humanized to HzKR127-3.2 and is currently in preclinical development^23^.

The human antibody we describe here was isolated by screening directly against live virus. This has the advantage of presenting the relevant viral antigens in the structural context in which they are displayed to the human immune system.

HuMAb006-11 was isolated from a convalescent patient by screening against HBV Genotype D. We show that HuMAb006-11 binds to a conformational epitope on HBsAg found on all four major genotypes and has potent neutralizing activity. Next, we engineered recombinant human IgG subclass variants of HuMAb006-11 to test the influence of isotype on binding and neutralizing activity. Whilst HuMab006-11 was isolated from the IgG1 subclass, the IgG4 subclass showed significantly better neutralizing activity. Finally, HuMAb006-11-IgG1 demonstrated superior prophylactic and therapeutic utility in vivo compared to HBIG. As a post-infection anti-viral agent, HuMAb006-11 significantly reduced HBV DNA levels and circulating HBsAg. These findings indicate that HuMAb006-11 represents a potential modality for medical intervention in HBV.

## Results

### HBV-specific human antibody isolation

**Fig. 1:**
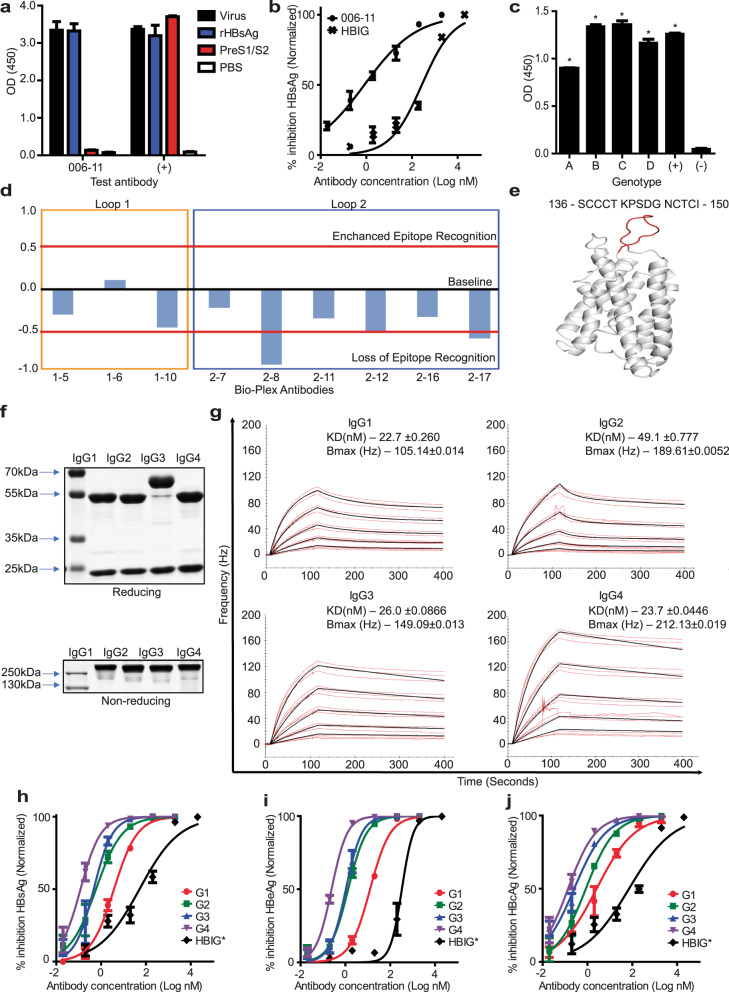
HuMab006-11 discovery and characterization. **a** HuMAb006-11 binding specificity was assessed at 5 μg/mL against live virus, recombinant HBsAg-S, and recombinant PreS1/2 protein. **b** The neutralizing potential of HuMAb006-11 & HBIG was tested via an in vitro assay utilizing HBV (genotype D) and the HepG2-hNTCP cell line. **c** The binding potential of HuMAb006-11 to the four most common HBV genotypes was tested by ELISA, statistical analysis was performed using the *t*-test, *p < 0.05 (Sequence data, Supplementary data—Table 1). All data were expressed as mean ± SEM, *N* = 4 independent experiments. **d** Binding of HuMAb006-11 to HBsAg virus-like-particles interferes with/reduces detection by Bio-Plex mAbs specific for the loop-2 regions (antibodies 8 and 17). A reduction >0.5-fold in comparison to the control indicates that the epitope recognition is substantially reduced for the tested bio-plex mAbs due to the binding of HuMab006-11. The *y*-axis reports ± fold-change with respect to the HBsAg standard with 95% CI ± 0.5-fold relative to the HBsAg standard. **e** Predicted HBsAg structural data (I-Tasser) with highlighted loop-2 region indicating the broad binding region of HuMAb006-11. **f** Heavy and light chains of HuMAb006-11 for the four principle IgG subclasses were resolved on polyacrylamide gel under reducing and non-reducing conditions. **g** Affinity and *B*_max_ measurements of HuMAb006-11 subclasses by QCM using recombinant HBsAg as target (mean ± s.e.m., *N* = 3 independent experiments). The dissociation equilibrium constant (*K*_D_) and *B*_max_ for each subclass are indicated at the top right-hand corner. Neutralization data comparing the four principle subclasses of HuMAb006-11 and HBIG based on **h** Secreted HBsAg, **i** secreted HBeAg. **j** intracellular HBcAg (representative flow data for quantification used to plot the HBcAg neutralization curve found in Supplementary Fig. 3c). All blots were derived from the same experiment and processed in parallel. All data were expressed as mean ± SEM, *N* = 3 independent experiments.

To facilitate the discovery of human anti-HBV antibodies, a capture ELISA allowing for an unbiased screen for human antibodies against the whole virus (Supplementary Fig. 1a–c) was developed. This assay was used in conjunction with PCR-based isolation of antibody heavy and light-chain genes from the identified HBV-specific memory B-lymphocytes derived from an HBV-resolved individual. A schematic of the discovery method and flow cytometry sorting strategy for memory B cells from PBMC’s is shown in Supplementary Fig. 1d, e. We selected a fully human IgG1 antibody termed HuMAb006-11, which bound to recombinant small-HBsAg (referred to as HBsAg) and HBV virions (Fig. 1a and Supplementary Fig. 2a) for further characterization.

The neutralizing potential of HuMAb006-11 was tested in vitro by employing HepG2/NTCP cells susceptible to HBV infection. HuMAb006-11 was found to be a potent neutralizer due to its ability to prevent HBV infection at nanomolar concentrations (Fig. 1b). HuMAb006-11 was observed to be superior to HBIG by >1000x when compared by EC_50_. Further examination of the cross-binding breadth of HuMmAb006-11 showed a similar binding profile to the four major genotypes (A, B, C, and D) of HBV (Fig. 1c, Sequences in Supplementary data—Table 1).

We observed that HuMAb006-11 lost binding activity when HBsAg was chemically reduced (Supplementary Fig. 2b). Further analysis by BioPlex assay showed that HuMAb006-11 blocked access to epitopes only in the loop-2 region (Fig. 1d) within the AGL domain in HBsAg. Additionally, when HuMAb006-11 was tested for its binding activity against the loop-1 (aa123–137 of HBsAg) and loop-2 (aa139–148 of HBsAg) cyclic peptides, it showed no binding activity (Supplementary Fig. 2c). To visualize the predicted binding interface for HuMAb006-11, a 3D structure of HBsAg Genotype A was predicted using the I-TASSER server, and the binding region/interface is highlighted in red (Fig. 1e). Collectively, these data suggest that HuMAb11 binds to a denaturation-sensitive, conformational epitope found on HBsAg.

### A functional comparison of HuMAb006-11-IgG subclasses

The variable region of the HuMAb006-11 heavy chain was engineered onto the backbone of the four principal human IgG subclasses. This was done to test for subclass-specific differences in the binding and neutralizing ability of HuMAb006-11. Figure 1f shows the reducing and non-reducing gels, of the four expressed subclasses. The presence of a single band on the non-reducing gel and two bands on the reducing gel of ~150 kDa and 50 kDa, 25 kDa, respectively, indicates that the antibodies were expressed appropriately. The larger heavy chain band seen for IgG3 is due to a longer hinge region compared to the other subclasses. The binding activities of the IgG2, IgG3, and IgG4 subclasses were verified against recombinant HBsAg via ELISA and showed similar binding to the original IgG1 subclass (Supplementary Fig. 2d).

A detailed biophysical analysis of the interaction between the four principal IgG subclasses of HuMAb006-11 with HBsAg was conducted using Quartz Crystal Microbalance (QCM) technology. The calculated dissociation equilibrium constant (*K*_D_) for the four molecules was 22.7 nM, 49.1 nM, 26.0 nM, and 23.7 nM for IgG1, IgG2, IgG3, and IgG4, respectively (Fig. 1g). Minimal differences in affinity were observed between IgG1, IgG3, and IgG4 while IgG2 showed a slightly lower affinity. The other biophysical parameter analyzed was the *B*_max_ value, which is 105.14 Hz, 189.61 Hz, 149.09 Hz, and 212.12 Hz for IgG1, IgG2, IgG3, and IgG4, respectively (Fig. 1g). The *B*_max_ value indicates the maximum number of antibody molecules bound to the antigen.

The neutralizing potential of the four subclasses was compared, a schematic of the assay is shown in Supplementary Fig. 3a. Figure 1h, i, and j compare the neutralizing potential of the four antibody subclasses by quantifying secreted HBsAg, HBeAg, and intracellular HBcAg (Supplementary Fig. 3a–c), respectively. All three data sets indicate that the HuMAb006-11-IgG4 subclass is the most potent neutralizer with an EC_50_ of 0.242 nM (HBeAg data). This was followed by mAb006-11-IgG3 (EC_50_–1.05 nM), IgG2 (EC_50_–1.325 nM) and finally IgG1 (EC_50_–12.63 nM). All the subclasses of mAb006-11 were also observed to have a more potent neutralizing potential compared to HBIG (EC_50 –_329.9 nM).

Analyzing the neutralization potential and biochemical characteristics of the different subclasses, HuMAb006-11 -IgG4 is the best neutralizer due to its high binding affinity and high *B*_max_. Comparing IgG2 and IgG3 that are the next best neutralizers with only a slight difference in neutralizing potential, we observed that IgG2 has a higher *B*_max_ but lower affinity while IgG3 has a slightly lower *B*_max_ but a higher affinity. This means that both these characteristics are essential in determining the neutralizing potential of the antibody, the *K*_D_ value determines how fast the antibody binds and how long it stays bound, while the *B*_max_ value determines the total number of antibody molecules decorated on the antigen target. Both these factors thus play an important role in deciphering the antagonizing potential of this antibody to block HBV infection.

### HUMAb006-11 as a prophylactic and therapeutic agent for HBV infection in vivo

**Fig. 2:**
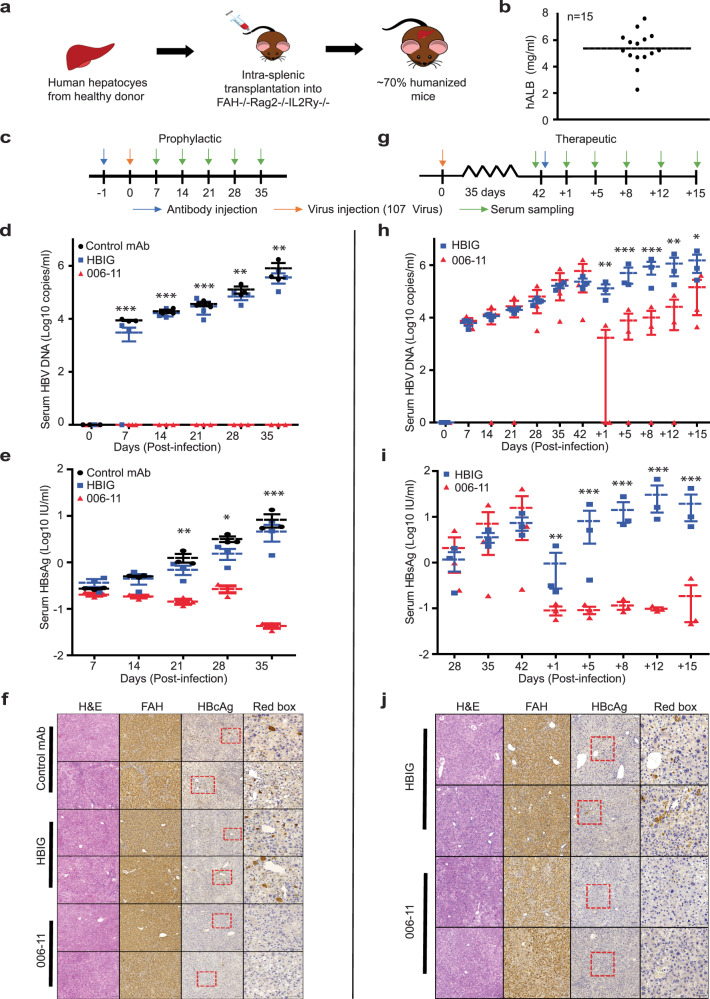
In vivo characterization of HuMab006-11. **a** Schematic representation of human liver chimeric mouse development. **b** Production of hALB in all mice was measured at ~5 mg/mL. **c** Schematic diagram indicative of antibody injection (HuMab006-11 (IgG1), virus inoculation, and serum sampling for prophylactic treatment. Weekly quantification of **d**. HBV DNA (statistical analysis between mAb006-11 and isotype Control mAb was performed using a *t*-test) and **e** HBsAg production (Statistical analysis between mAb006-11 and Control mAb was performed using an *F*-test) in mice injected with isotype control mAb, HBIG, or HuMAb006-11 one day before HBV inoculation of up to 35 dpi. **f** Histological analysis of mouse liver sections stained with hFAH, HBcAg, and H&E. **g** The schematic diagram for therapeutic treatment is indicative of antibody injection, virus inoculation, and serum sampling. Weekly quantification of **h**. HBV DNA (statistically analysis between HuMAb006-11 and HBIG was performed using an *F*-test) and **i** HBsAg production (statistically analysis between mAb006-11 and HBIG was performed using an *F*-test) in mice injected with isotype control mAb, HBIG, or HuMAb006-11 at 42 dpi following the establishment of HBV infection. Further quantification was performed at 1–4 days post-antibody injection intervals for 15 days. **j** Histological analysis of mouse liver sections stained with hFAH, HBcAg, and H&E. Scale bars for hFAH, HBcAg, and H&E are 100 µM. The scale bar for red box images is 50 µM. Each group composed by *n* = 3 mice *p < 0.05, **p < 0.005, ***p < 0.0005.

The prophylactic and therapeutic potential of HuMAb006-11 (IgG1) were investigated in a human liver chimeric (HuFRG, ~70% of human hepatocytes) mouse model^24^, which supports HBV infection. A schematic illustration of the humanization of HuFRG mice is shown in Fig. 2a. Mice are generally considered susceptible to HBV infection when hALB levels reach ~5 mg/mL, all mice used for this study had hALB levels ranging between 2–7 mg/mL, as shown in Fig. 2b.

We first evaluated the prophylactic efficacy of HuMAb006-11 in HBV-infected HuFRG mice. A single dose (3.3 mg/kg per mouse) of isotype control HuMAb or HuMAb006-11 was administered intraperitoneally to each mouse one day before HBV infection followed by measurement of HBV DNA weekly up to 63 days post-infection (dpi). Both mouse groups did not exhibit any physical abnormalities or changes in body weight (Supplementary Fig. 4b) post-infection, indicating that they remained healthy. However, significant differences in HBV DNA and serum HBsAg levels were observed between the two groups. The treated group (HuMAb006-11) displayed HBV DNA levels below the limit of detection whereas the control mAb group exhibited increasing DNA levels of up to ~9Log copies/mL (Supplementary Fig. 4c). Moreover, mice treated with HuMAb006-11 displayed HBsAg levels ~3Logs IU/mL lower at the experimental endpoint compared to the isotype control group indicating a complete blockade of viral entry (Supplementary Fig. 4d).

Next, we investigated the effects of HuMAb006-11 as a post-infection, anti-viral treatment. HBV infection was first established in HuFRG mice for 42 dpi before the administration of a single dose (10 mg/kg per mouse) of isotype control HuMAb or HuMAb006-11. Both HBV DNA and HBsAg levels were measured at 1, 4, and 8 days post-antibody treatment. At the endpoint, a ~3Log and ~2Log reduction was observed in HBV DNA and HBsAg levels respectively in mice injected with HUMAb006-11 compared to the isotype control group (Supplementary Fig. 5b, c).

Having observed potent prophylactic and post-infection anti-viral efficacy of HuMAb006-11 in vivo, we next compared the prophylactic efficacy of a single dose of HuMAb006-11 against HBIG versus an isotype control mAb. A single antibody dose (3.33 mg/kg per mouse) was administered intraperitoneally to HuFRG mice one day prior to challenge with HBV (Fig. 2c). Nine HuFRG mice were randomly injected with either a control HuMAb, HBIG, or HuMAb006-11 at identical concentrations and monitored over 35 days. Both the HBIG and control groups showed a steady increase in HBV DNA for 35 dpi (~6Log copies/mL) whereas DNA levels remained undetectable in the HuMAb006-11 treated group (Fig. 2d). In addition, serum HBsAg levels for both control mAb and HBIG groups were measured at around 1 log IU/mL, which is ~100 times higher compared to mice treated with HuMAb006-11 at 35 dpi (Fig. 2e). At the endpoint, mice liver, spleen, and kidneys were harvested for histological analysis. All mice liver sections stained positive for human fumarylacetoacetate hydrolase (hFAH) by immunohistochemistry (IHC) indicating a robust repopulation of human hepatocytes (Fig. 2f). Furthermore, intracellular HBcAg was detected (evidence of active infection) by IHC in liver sections of both control mAb and HBIG groups but not in HuMAb006-11-treated mice. Lastly, H&E staining of all control and treated mouse liver (Fig. 2f), spleen and kidney sections (Supplementary Fig. 4e) was carried out and did not exhibit any morphological abnormalities. This data demonstrates the prophylactic efficacy of HuMAb006-11 in preventing the establishment of an active infection.

Next, the therapeutic potential of HuMAb006-11 against an established infection was evaluated (Fig. 2g). Six mice measuring an average of ~5Log copies/mL HBV DNA at 42 dpi were randomly administered with a single dose (10 mg/kg per mouse) of the respective antibodies. Although mice treated with HBIG showed a small decrease in HBV DNA and HBsAg levels after 24 h, both levels rebounded to initial levels at 5 days post-injection and continued to rise over time (Fig. 2h, i). In contrast, HUMAb006-11-treated mice exhibited a robust decrease in both HBV DNA (~2Log copies/mL) and HBsAg levels (~2Logs IU/mL) after 1 day post-injection, suggesting that mAb006-11 is more efficient at blocking HBV replication than HBIG (Fig. 2h, i). Moreover, virus titer rebound was kept at a minimum level, whereas HBsAg production was maintained at close to undetectable levels (Fig. 2h, i). Similarly, histological analysis of mice liver sections in both groups displayed strong hFAH staining. In contrast, HBcAg staining was more abundant in liver sections of HBIG treated mice compared to HUMAb006-11 (Fig. 2j). H&E staining of mice liver, kidney, and spleen sections did not exhibit any morphological abnormalities (Fig. 2j and Supplementary Fig. 5d).

## Discussion

In this study, we describe the isolation, characterization, and in vivo testing of a fully human monoclonal antibody with potent neutralizing efficacy. HuMAb006-11 was isolated directly from a convalescent patient by screening against the live virus. Its heavy and light chains belonged to the IgG1 subclass and Lambda light-chain group. In this study, direct screening against the whole virus for isolation of naturally paired, fully human anti-HBs antibodies was conducted. The screening methodology potentially allows for the non-biased isolation of antibodies against all three surface antigens in their native state found on the surface of the virus. This is essential as structural analyses to define the differences between the presentation of HBsAg on recombinant sub-viral particles and virions have not been conducted. In Fig. 1c, we present the neutralization potential of HuMAb006-11 and show its superiority compared to HBIG at preventing HBV infection in vitro. HuMAb006-11 displayed a cross-binding activity for the four most common HBV genotypes (A, B, C, D) indicating that it is binding to a conserved epitope.

Subclass engineering was carried out to determine if structural differences between subclasses affect HuMAb006-11’s binding and neutralizing potency. Subclass differences had little impact on the binding affinity of HuMAb006-11 to HBsAg as defined by their nanomolar dissociation constant (Fig. 1g) for the three most commonly seen subclasses. The *B*_max_ values for the subclasses differed significantly suggesting that structural differences are affecting the stoichiometry of antibody binding to HBsAg. The two smallest IgG subclasses (IgG2, IgG4) showed the highest *B*_max_ values followed by IgG3, which has a highly flexible hinge then IgG1. The neutralizing potential of the four subclasses was noted to be in the following order IgG4, IgG3, IgG2, and IgG1 (Fig. 1h–j). Neutralizing potency closely correlates with the *B*_max_ value and to some degree affinity, indicating that both parameters play an essential role in this functional activity. Previous studies analyzing anti-HBs subclasses have reported that IgG1 and IgG3 levels are the most dominant followed by IgG4 in convalescent and chronic-infected patients^25^. Other studies have intimated that anti-HBs IgG4 is found in immune complexes that are cleared inefficiently due to a lower affinity for Fc receptors^26^. This potentially suggests that the IgG4 antibody response seen in chronic patients might be a protective mechanism aimed at reducing the virus-driven inflammatory response. The more potent neutralizing effect observed for HuMAb006-11-IgG4 can potentially be attributed to IgG4 molecules decorating the surface of virions more efficiently, thus preventing virus-receptor attachment and infection. This hints that IgG4 antibodies could function efficiently as antagonists, preventing viral infection and spread. The indication is that while IgG1 and IgG3 antibodies play an important role in activating downstream effector functions to clear the virus in acute or chronic infection, IgG4 subclasses may be the best choice for prophylactic approaches.

We examined the prophylactic potential of HuMAb006-11 against HBV in a human liver chimeric mouse model. Since wild-type mice cannot support human-specific HBV infection due to the lack of the relevant NTCP receptors, the HuFRG mice model offers the best in vivo representation of an active infection in humans. We present evidence showing that a single dose of HuMAb006-11 effectively prevented HuFRG mice from establishing an active HBV infection. Our in vivo data indicates that HuMAb006-11 potently inhibits viral entry and also affects the clearance of virions from circulation. When compared with HBIG, the current antibody-based prophylactic modality used clinically, HuMAb006-11 showed superior protection. Moreover, when used as a therapeutic intervention strategy, HuMAb006-11 showed a potent reduction in both HBV DNA and serum HBsAg levels of 2–3logs copies/mL and IU/mL, respectively. Most importantly, the antibody dosage of HuMAb006-11 needed was significantly lower (3 mg/kg for prophylactic and 9 mg/kg for therapeutic) compared to other reported anti-HBs antibodies (15–20 mg/kg for prophylactic and 20 mg/kg for therapeutic with multiple doses) in vivo^9,14^, which further supports our conclusion that HuMAb006-11 specifically targets an essential epitope required for viral entry, thus effectively inhibiting infection.

Finally, the recombinant, monoclonal nature of HuMAb006-11 represents an additional advantage compared to HBIG, which is derived from purified human plasma and is plagued by issues such as limited availability, low specificity, and batch to batch variation. In contrast, a monoclonal anti-HBsAg should provide a stable and reproducible source for future clinical employment. Taken together, these data have important implications for our understanding of the fundamental biology of how human antibodies can be mobilized to target HBV and indicate that HuMAb006-11 is a good candidate for future clinical development.

## Methods

### Ethics statement

Human peripheral blood was obtained after informed consent from an acute-recovered HBV patient (DSRB 2015/00354, Hepatitis B virus eradication and loss (HEAL) cohort study). Study protocols were approved by the National University of Singapore Institutional Review Board. Participants were recruited after providing written informed consent to take part in the study under a DSRB approved protocol. All procedures performed involving human participants complied with relevant ethical regulations. The International Animal Care and Use Committee (IACUC), A*STAR specifically approved this study under the protocol number #181417. All animal experimental procedures were conducted in accordance to IACUC’s guidelines.

### Whole antibody or antibody fragment expression

Heavy- and light-chain variable regions of the selected antibody were cloned into plasmids with the respective human IgG (IgG1, IgG2, IgG3, IgG4) constant regions. Plasmids were transformed into E.coli TOP10 by heat shock. A single colony was picked and grown overnight before plasmid extraction using E.Z.N.A endo-free plasmid DNA mini kit (Omega Bio-Tek, cat. #D6950-01). Sequencing was performed to ensure no mutations were introduced. Transfection of heavy and light-chain plasmids into HEK293 cells with polyethyleneimine (Sigma-Aldrich, cat #764965) was performed for the expression of full-length antibodies. Antibodies were purified from the culture supernatants using protein G Sepharose 4 fast flow resin (Cytiva, cat. #17061805).

### Antibody quantification

To measure the concentration of purified antibodies, the Bradford method was used. In all, 200 µL of protein assay reagent (Bio-Rad Protein Assay Dye Reagent Concentrate, Cat. 5000006) was mixed with 10 µL of antibody sample or antibody IgG standards in a flat-bottom 96-well plate. The mixture was incubated for 10 min at room temperature and read at an optical density of 595 nm on a microplate reader. An IgG standard curve (Pierce™ Bovine Gamma Globulin Standard Ampules, Cat 23212) was generated to determine protein concentrations.

### HepG2-hNTCP cells

HepG2-hNTCP cells were cultured in Dulbecco’s Modified Eagle Medium (DMEM) (Sigma-Aldrich, cat #D5671) + 10% fetal bovine serum (FBS) (Gibco, cat. #26140) + 2.5% puromycin in a T75 flask and maintained at 37 °C with 5% CO_2_ in a cell incubator.

### Virus production

To produce HBV virus for infection, HepAD38 cells^27^ (Genotype D) were grown in a T75 flask and cultured in DMEM medium with tetracycline at 37 °C with 5% CO_2_ in a cell incubator. After 2 weeks, the medium was changed to DMEM medium without tetracycline (tet off). The cell supernatant was collected every three days. After collection, the virus was concentrated using 8% PEG 8000 before resuspension in PBS/10% FBS at 1/100 the volume. The virus mixture was aliquoted and stored at −80 °C for use.

### Capture ELISA

Briefly, Maxisorp plates were coated with a capture antibody (Fitzgerald, PreS2, Cat. 10-H08A) at 5 µg/mL overnight. Plates were blocked with 4% skim milk in PBS (SM-PBS) buffer at room temperature for 2 h and then incubated with the virus at 4 °C overnight. Plates were then incubated with antibodies at room temperature (various concentrations) diluted in blocking buffer for 1 h. Each well was then incubated with an HRP-conjugated goat-anti-human IgG secondary antibody (Thermo Fisher, cat. #31413, diluted 3000×) for 1 h, followed by incubation with TMB substrate (Thermo Fisher, cat. #34028) for color development before H^2^SO^4^ addition to stop color development. OD450 was recorded. Plates were washed four times with PBS between all incubation steps. Antibody-coated wells with no virus added were included as negative controls.

### Sorting of memory B Cells

Cryopreserved human PBMCs were thawed at 37 °C in RPMI/10% FBS. Cells were washed with PBS, stained with Live/Dead blue viability dye (Thermo Fisher, Cat. L23105), and incubated in a cocktail of monoclonal antibodies (CD19—1:50, CD14—1:50, CD3—1:100, CD27—1:50, CD38—1:50, IgG—1:50, IgM—1:50, Refer to Supplementary Table 2 for manufacturer/catolog details). B cells were washed in PBS and small pools of 20 cells were sorted into 384-well plates using the BD FACSAria III cell sorter.

### B-cell screening

Isocove’s Modified Dulbecco’s Medium (IMDM) + Glutamax (Gibco, cat. #31980-030) supplemented with 10% ultra-low IgG FBS (Gibco, cat. #16250-078) and 1% penicillin/streptomycin (Gibco, cat. #15140-122), 50 µg/mL human transferrin (Sigma-Aldrich, cat. # I9278), and 5 µg/mL human insulin (Sigma-Aldrich, cat. # T8158), was used for culturing of human memory B cells. The medium is referred to as complete IMDM. The sorted human memory B cells were then resuspended in complete IMDM with the addition of an activation cytokine milieu, containing the following: 20 U/mL IL-2 (Cell Guidance Systems, cat. #GFH12), 50 ng/mL IL-10 (Cell Guidance Systems, cat. #GFH83), and 10 ng/mL IL-15 (Cell Guidance Systems, cat. #GFH86) and 50 ng/mL monomeric soluble recombinant human CD40L (eBioscience, cat. #34-8902-81). The memory B cells were cultured for 4 days under these conditions to allow for stimulation and expansion. After 4 days, the cells were pelleted and resuspended in new complete IMDM with the addition of a secretion cytokine milieu, containing the following: 20 U/mL IL-2, 50 ng/mL IL-10, 10 ng/mL IL-15, and 50 ng/mL IL-6 (Cell Guidance Systems, cat. #GFH10). This was to promote differentiation to antibody-producing plasmablasts. Following another 3 days of culture, the cells were pelleted, supernatants used for ELISA screening, and pellets lysed using the QuickExtract RNA extraction kit (Lucigen). The supernatants were harvested and used for ELISA screening. All cytokines were purchased from Cell Guidance Systems, USA.

### Antibody sequence recovery and synthesis

Cell lysates, corresponding to the positive hits from the ELISA screen, were then used for downstream PCR and NGS analysis. cDNA was generated using the Maxima H Minus cDNA Synthesis Master Mix (Thermo Fisher, Cat. #K1681) using 5 μL of extraction buffer per 10 μL reaction as per the manufacturer’s protocol. Two microliters of cDNA products were directly used in 20 μL reactions with Platinum Taq Master Mix as per the manufacturer’s protocol for separate amplification of the heavy and light-chain variable regions. Illumina adaptor and barcode sequences were added by further PCR with Q5 Hot Start Hi-Fidelity Master Mix and the PCR products were purified with AMPure XP magnetic beads. Purified PCR products were pooled to obtain a 4 nM library and sequenced on the Illumina MiSeq with a 2× 300 bp kit with 25% PhiX spike-in. Individual reads were separated and corresponding germline sequences were retrieved from IMGT, the international ImMunoGeneTics database. The individual heavy and light sequences obtained were sent to Twist Biosciences for plasmid synthesis.

### Sodium dodecyl sulfate-polyacrylamide gel electrophoresis (SDS-PAGE)

Gel electrophoresis was carried out with a 12%/15% polyacrylamide resolving gel to visualize protein sizes under reducing or non-reducing conditions. In all, 5 μg of protein mixed with reducing or a non-reducing dye, incubated at 95 °C for 10 min was loaded onto separate lanes. Protein ladder (Thermo Fisher, cat. #26619) was loaded as a reference. Coomassie Brilliant Blue was used for gel staining before imaging on a gel imager (Bio-Rad). The unprocessed figures can be found in the supplementary information file.

### Western blotting

Briefly, protein bands separated on SDS-PAGE were transferred onto a polyvinylidene fluoride (PVDF) membrane. The membrane was blocked in 5% skim milk in PBST for 1 h, before incubation with 10 μg/mL primary antibody or control antibody. Next, the membrane was incubated with goat-anti-human IgG-HRP (Thermo Fisher Scientific, cat. #31413, diluted 10,000×). Three washes for 10 min each in PBST were conducted after step. WesternBright ECL (Advansta, cat.#K12045) was added for protein visualization in the darkroom. Images were taken with an X-ray film (Advansta, cat.#L-07014-100). The unprocessed figures can be found in the supplementary information file.

### HBV neutralization assay

Briefly, 5 × 10^4^ HepG2-hNTCP cells were seeded into a 96-well plate, 1 × 10^3^ GE/cell of HBV and the respective antibody was mixed and inoculated with HepG2-hNTCP cells in the presence of 4% PEG 8000 (Sigma-Aldrich, cat. #729108). After 20 h of incubation at 37 °C, with 5% CO_2_, cells were thoroughly washed three times with 1x PBS and maintained in infection medium. Medium change was performed every 2 days. On day 7, supernatant from individual wells was harvested and used for the analysis of secreted HBsAg (Bio-Rad–Monalisa HBsAg kit cat. #72346) and HBeAg (BioRad–Monalisa HBeAg kit, cat. #25220). The cells were harvested by trypsinization for analysis of intracellular HBcAg (Thermo Fisher, cat. #MA1-7606) via flow cytometry.

### Flow cytometry (HBV HBcAg)

Harvested HepG2-hNTCP cells were fixed and permeabilized using the fixation/ permeabilization solution kit (BD Biosciences cat. #554714) following kit instructions. Cells were incubated with HBcAg (Thermo Fisher Scientific, cat. #MA1-7606) at 1000x dilution at 4 °C for 1 h in permeabilization buffer. After washing, cells were stained with AlexaFluor-647 conjugated goat-anti-mouse IgG antibo\dies (Invitrogen cat. #A21235) at a 300x dilution. Uninfected cells were stained with the same protocol as a control. All sample data were acquired using the Attune NxT flow cytometer and data analyzed using FlowJo software.

### Affinity determination of antibodies

Antibody kinetics and affinity were measured using an Attana Cell A200 (Attana AB) at 25 °C. An LNB carboxyl sensor chip was used for the experiments. (Attana AB, cat #3623-3033) Activation of the chip was carried out with sulfo NHS/EDC (Amine coupling kit, Attana) and only the experimental chip was saturated with rHBsAg. Ethanolamine was added to deactivate the chip surface. PBS was used as a running buffer for subsequent injections. Antibody dilutions were optimized together with regeneration conditions and randomly tested by the robotic arm. Antibody injections were applied as 105 s pulses at a flow rate of 20 μL/min and dissociation was monitored for 300 s. Regeneration of the chip was done by two 10 s pulses of 20 mM Glycine, pH 5.0. Curve fitting and data analysis were performed using Trace Drawer software.

### HBV DNA quantification

HBV DNA was isolated using the Qiagen QIAamp DNA Blood Kit (Qiagen, cat. #51104). Briefly, lysis buffer together with Proteinase K was added to the sample at 57 °C for 15 min. The sample was spun through the provided column and washed thoroughly, before elution. 5 µL of the eluted sample was used for qPCR. qPCR was performed with an SYBR Green qPCR (Thermo Fisher, cat. #4309155) using HBV DNA-specific primers: (1) CCTGGTTATCGCTGGATGTGT and (2) GGACAAACGGGCAACATACCTT using ABI7500 Fast Real-Time system instrument (Applied Biosystems). Viral DNA copy number was calculated based on a standard curve generated from a sample with known copy numbers.

### Immunoprecipitation assay

In all, 100 µL of HBV was incubated with an isotype control antibody and protein G Sepharose for 2 h at 4 °C for pre-clear. The sample was divided and immunoprecipitated with the antibody of interest, positive control (Fitzgerald PreS2 antibody), or PBS (negative control) overnight at 4 °C. Next, protein G beads were added and rotated for 2 h at 4 °C. The beads were spun down washed three times with 0.1% PBST. HBV DNA extraction was performed for quantification.

### Multiplex immunoassay

Using the Bioplex 200 platform (BioRad), a multiplex HBsAg epitope mapping was developed targeting anti-HBs epitopes across five HBsAg domains^12,28^. Individual fluorescently identified magnetic beads were preconjugated with anti-HBs mAbs and plexed together. Polyclonal phycoerythrin-conjugated antibodies were used for detection. The epitope specificity of the selected multiplex anti-HBs mAbs to particular HBsAg domains spans residues 99–160 in the “a” determinant and targets the loop-1 and loop-2 regions. Using this method, the effect of anti-HBs antibodies on the HBsAg epitope profile was investigated by pre-incubating HuMAb11 with an HBsAg wildtype reference, prior to testing in the multiplex immunoassay. On analysis, alterations in the epitope profile mapping between HBsAg only and HBsAg pre-incubated with HuMAb11 are compared.

The conjugated mAbs have the following specificities, mAbs 5, 6, and 10: loop-1; mAbs 7, 8, 11, 12, 16, and 17: loop2. The origin of the mAbs is acknowledged by Hyakumura et al.^28^, and an illustration of the antibody binding sites has been published by Walsh et al.^12^. The 95% confidence interval (CI) for the normal range of variation of epitope recognition from the reference backbone was established as ±0.5-fold-change. A fold-change <0.5 was considered insignificant reflecting the normal variance of this assay. Positive fold-changes (>0.5-fold) and negative fold-changes (>0.5-fold) corresponded to a gain or reduction of epitope binding, respectively. A threefold reduction was considered a complete epitope knockout.

### Epitope binding

A direct ELISA protocol was carried out coating HBsAg peptides (50 ng/well): (loop-1, T-C-T-T/I-P-A-Q-G-N/T-S-M-F-P-S-C, loop-2, C-T-K-P-T/S-D-G-N-C-T) in phosphate-buffered saline (PBS). These were bound to microtiter plates (Maxisorp, Nunc) at 4 °C overnight, and then each well was blocked in 5% skim milk in PBS Tween 20 (0.05%) at room temperature for 2 h. Antibodies (mAb11) were incubated at an appropriate dilution in PBS for 1 h at room temperature. Bound antibody was detected with an anti-human immunoglobulin (Ig) conjugated to horseradish peroxidase. After several washing steps, antibody binding was detected by the addition of ABTS [2, 2’-azinobis (3- ethylbenzthiazoline-6-sulfonic acid); Sigma] and H_2_O_2_ in a citrate phosphate buffer. OD’s were read at 410 nm after 30 min.

### Generation of human liver chimeric mice

Fah^-/-^/Rag2^-/-^/IL2rg^-/-^(FRG) triple knockout mice purchased from Yecuris Corporation were maintained with (2-(2-nitro-4-trifluoromethylbenzoyl)-1,3-cyclohexanedione) (NTBC) at a concentration of 16 mg/mL. All mice were bred and kept under specific pathogen-free with 12-h light/12-h dark cycle conditions in the Biological Resource Centre, Agency for Science, Technology and Research, Singapore in accordance with the guidelines of the Agri-Food and Veterinary Authority and the National Advisory Committee for Laboratory Animal Research of Singapore. Briefly, 4-6-week-old mice were intrasplenically injected with 1 × 10^6^ primary human hepatocytes (PHH) (Lonza, Cat. HUCSD). Following surgery, NTBC was withdrawn through a series of cycling conditions to facilitate mouse hepatocyte cell death. Repopulation of human hepatocytes generally takes around 2–3 months whereby the production of ~5 mg/mL human albumin (hALB) in mouse serum represents ~70% humanization of the liver indicative of mice being experimentally ready for HBV infection.

### Quantification of hALB production

Production of hALB in mice was measured by ELISA according to the manufacturer’s instructions (Bethyl Laboratories Inc). Briefly, serum isolated from mouse blood was diluted 10^6^ times with sample diluent followed by quantifying hALB using the kit’s protocol.

### Quantification of HBV DNA levels in mouse serum

To determine serum HBV DNA levels, 1 µL of serum was diluted in 9 µL of water. The mixture was then incubated for 10 min at 100 °C, after which 2 µL was used as the qPCR template for DNA quantification. The qPCR was performed with SsoFast EvaGreen Supermix (Bio-Rad, cat.#1725200AP) using HBV-specific primers: (1) CCGTCTGTGCCTTCTCATCTG and (2) AGTCCAAGAGTCCTCTTATGTAAGACCTT using the ABI7500 Fast Real-Time system instrument (Applied Biosystems). The viral DNA copies were calculated based on a standard curve generated from samples with known DNA copy numbers.

### Quantification of HBsAg in mouse serum

Mice serum was diluted 10x in PBS before quantification of HBsAg according to the manufacturer’s instructions (Autobio, HBsAg CLIA, cat. #CL0310-2). Briefly, serum from different time points was incubated with an enzyme conjugate in a pre-coated ELISA plate for an hour. Six washes were performed with PBS followed by the addition of a substrate cocktail provided. HBsAg was detected by luminescence using a Tecan plate reader. The kit also provides a series of HBsAg standards in International Units per mL (IU/mL) to generate a standard curve for HBsAg quantification.

### Histological analysis of mouse organs

All mouse livers, kidneys, and spleens were collected and processed for histology at the endpoint. Briefly, all tissues were fixed in 10% formalin before paraffin-embedment. All tissue sections were rehydrated and stained with H&E (ThermoFisher) according to the manufacturer’s protocol. Liver sections were also stained with hFAH (Abcam, cat. #ab14016) and purified recombinant HBcAg (ThermoFisher, cat.#MA1-7607) antibodies by immunohistochemistry according to the manufacturer’s protocol. All images were captured by the ZEISS Axio Scan.Z1 and processed using the Zen lite software.

### Reporting summary

Further information on research design is available in the Nature Research Reporting Summary linked to this article.

## Supplementary information

Supplementary Figure and Tables

REPORTING SUMMARY

## Data Availability

The data that support the findings of this study are available from the corresponding author upon reasonable request.
