# Supplementary material for: Defining the specificity and function of a human neutralizing antibody for Hepatitis B virus
Source: NPJ Vaccines. 2022 Oct 21;7:121. doi: 10.1038/s41541-022-00516-7 (PMC9586962; doi:10.1038/s41541-022-00516-7)
Supplement: Supplementary file 1 — Supplementary Figure and Tables [file 41541_2022_516_MOESM1_ESM.pdf]

Supplementary Figures and Tables

Supplementary Figure 1

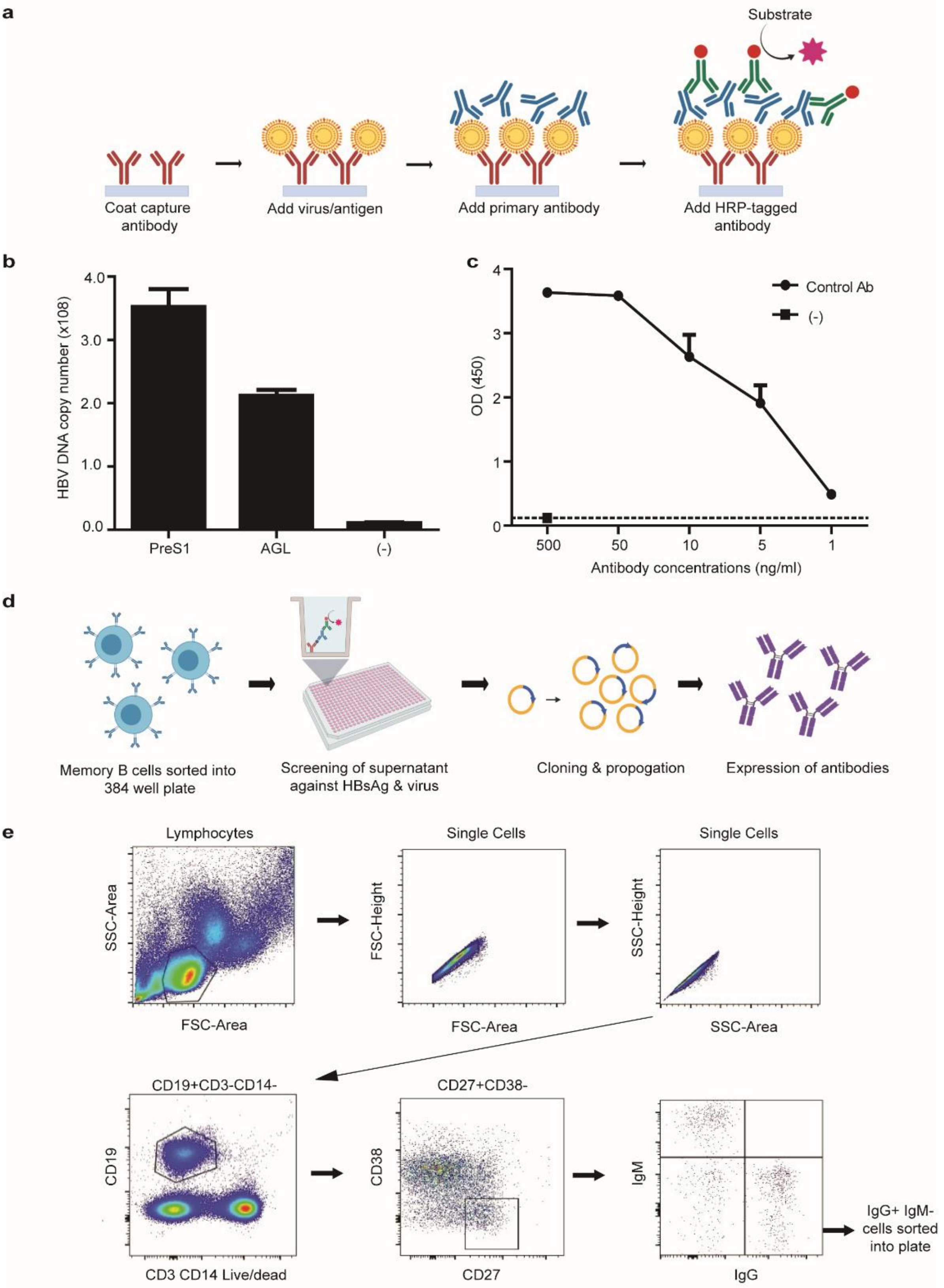

Development and optimization of capture ELISA for live virus screening and antibody discovery methodology. **a.** A schematic representation of the ELISA assay. **b.** Testing of two commercial mouse antibodies (anti-preS1, anti-AGL) for determining the capture

antibody reagent. Antibodies were compared by immunoprecipitation to determine which antibody captures the virus most effectively based on HBV genome copy number comparison. **c.** Optimization of the capture ELISA, with a primary antibody (human) at varying concentrations and a secondary goat anti-human antibody. **d.** Schematic representation of antibody discovery methodology. **e.** Flow cytometric data for the selection of memory B cells for high-throughput antibody discovery

Supplementary Figure 2

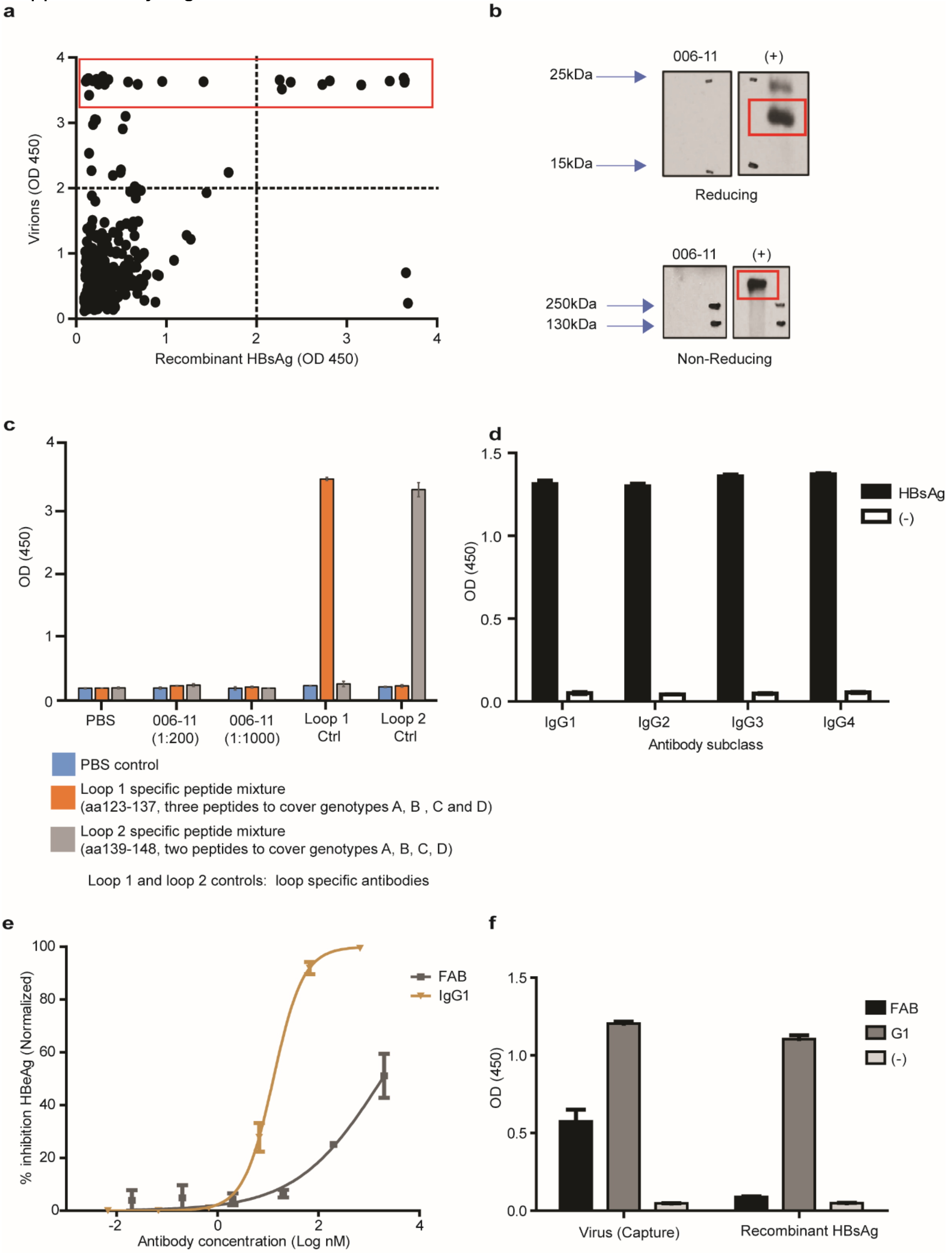

Discovery, binding characterization, and subclass engineering of HuMAb006-11 a. Isolation of HuMAb006-11 was done by performing a double screen against live virus and recombinant HBsAg. The wells containing cells within in red box were picked for sequence recovery. **b**. Immunoblot detection of HuMAb006-11-IgG1 binding recombinant HBsAg under reducing (SDS + 2-Beta mercaptanol) and non-reducing (SDS only) conditions. Commercial goat anti-HBs (polyclonal) was used as a positive control. The protein ladder is labelled. **c**.

Testing of HuMAb006-11 binding to linear peptides of HBsAg loop 1 and loop 2 regions. The medium value of two independently performed assays is shown. Results were deemed positive if the optical density (OD 450nm) of the medium value was above the OD of the negative control plus 2 standard deviations. This indicates that HuMAb006-11 is binding to a conformational epitope. **d.** Binding specificity of four principle IgG subclasses of HuMAb006-11 at 5 μg/ml to recombinant HBsAg was tested by ELISA (mean ± SEM, *N* = 4 independent experiments) **e,f.** mAb006-11 FAB and IgG1 versions were compared for binding and neutralization efficacy. The FAB version of mAb 006-11 showed meaningfully lower binding and neutralizing potential compared to the IgG1 version. This indicates that mAb006-11 requires bivalency to affect its functional activity.

Supplementary Figure 3

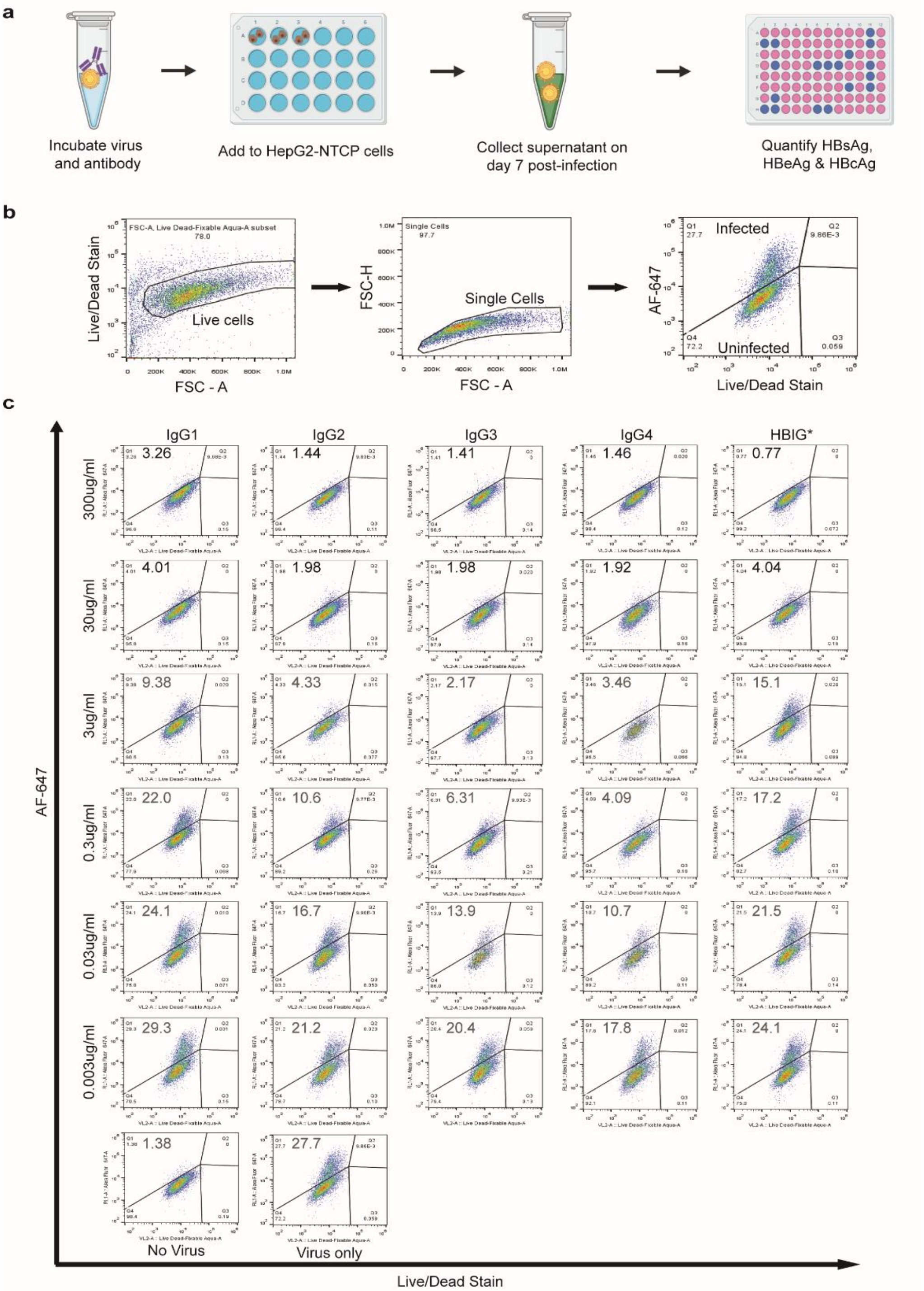

Gating strategy and measurement of intracellular HBcAg by flow cytometry. **a**. Schematic representation of in-vitro neutralization assay. **b**. Gating strategy used for quantification of intracellular HBcAg on Day 7 post-infection. HepG2-hNTCP infected cells were quantified by staining for $BV510^-$ (live/dead stain) and $AF647^+$ (Goat-anti Mouse secondary antibody binding to mouse anti-HBcAg). **c**. Flow plots for the various concentrations of the four IgG subclasses of HuMAb006-11 and HBIG, no virus, and virus only wells (representative plot from one experiment is shown). *HBIG concentration was 10x higher (3000ug/ml, 300ug/ml,30ug/ml, 3ug/ml, 0.3ug/ml, 0.03ug/ml)

Supplementary Figure 4

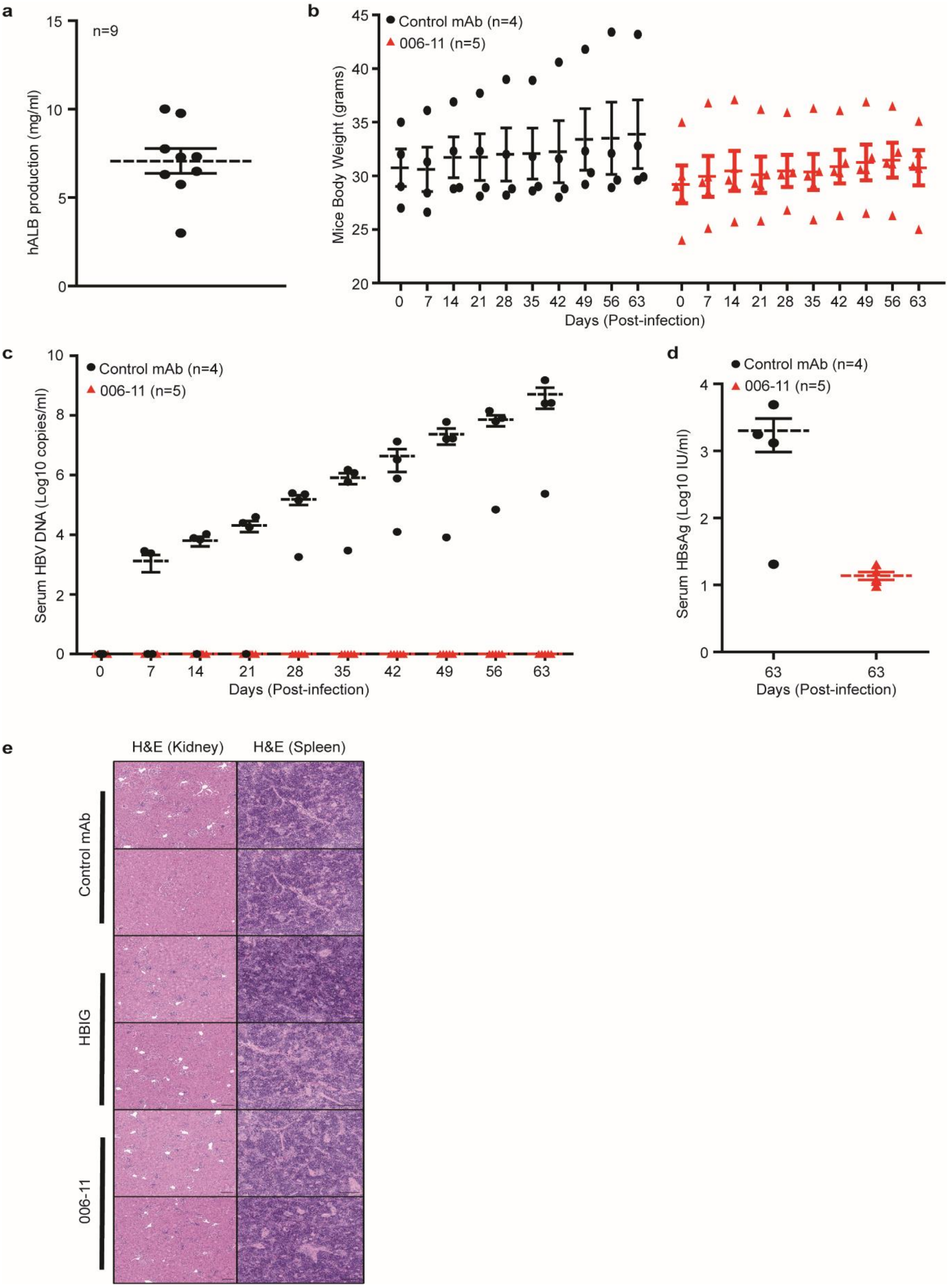

Prophylactic validation of HuMAb006-11 *in vivo*. a. Production of hALB in all mice was measured at ~7mg/ml. Weekly **b.** body weight (g) changes and **c.** HBV DNA quantification in mice injected with isotype control mAb or HuMAb006-11 one day before HBV inoculation of

up to 63 dpi. **d.** Measurement of HBsAg production in all mice at 63 dpi. **e.** Histological analysis of mouse kidney and spleen sections stained with H&E. Scale bars for H&E images is 100uM

Supplementary Figure 5

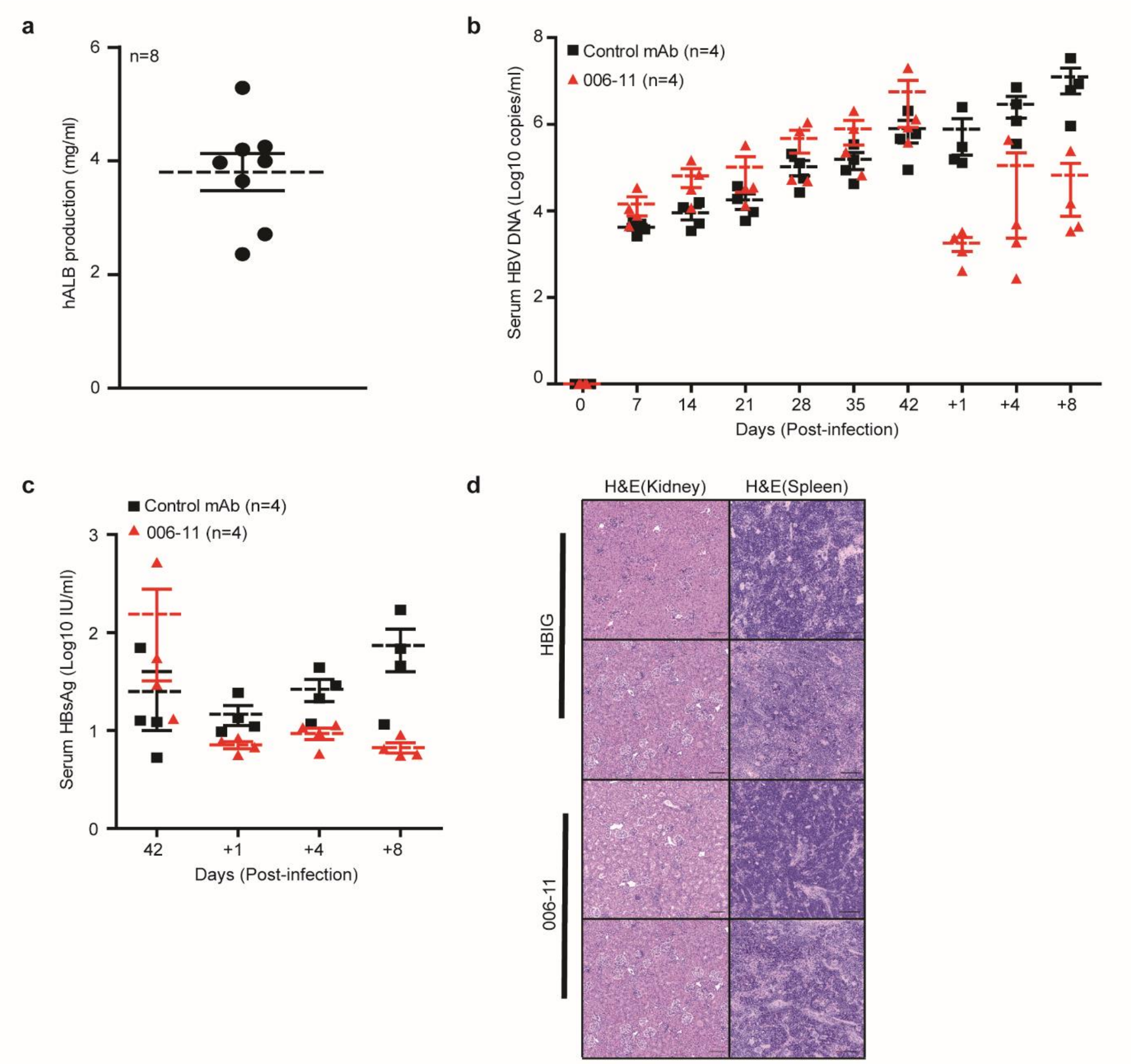

Therapeutic validation of HuMAb006-11 *in vivo*. **a.** Production of hALB in all mice was measured at ~4mg/ml. **b.** Weekly quantification of HBV DNA in mice injected with isotype control mAb or HuMAb006-11 at 42 dpi following the establishment of HBV infection. Further quantification was performed at 1-3 days post-antibody injection intervals for 8 days. **c.** HBsAg production was measured at 42 dpi followed by 1-3 days post-antibody injection intervals for 8 days. **d.** Histological analysis of mouse kidney and spleen sections stained with H&E. Scale bars for H&E images is 100uM

Supplementary Figure 6

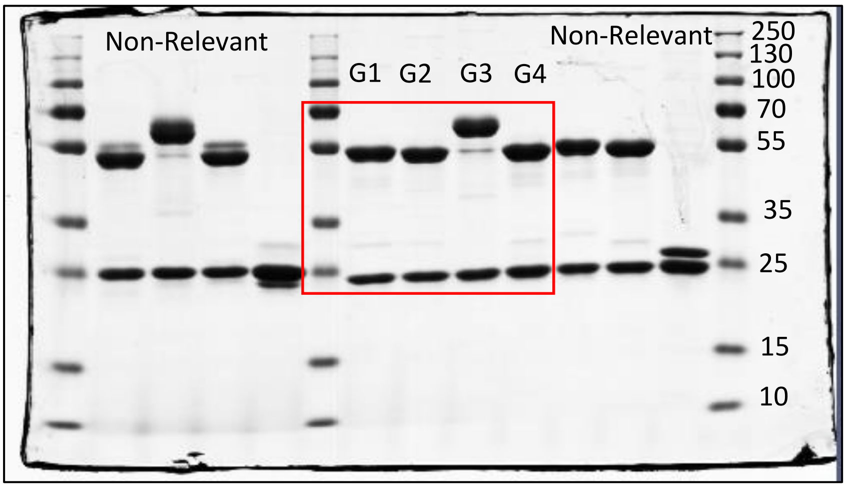

Reducing Gel

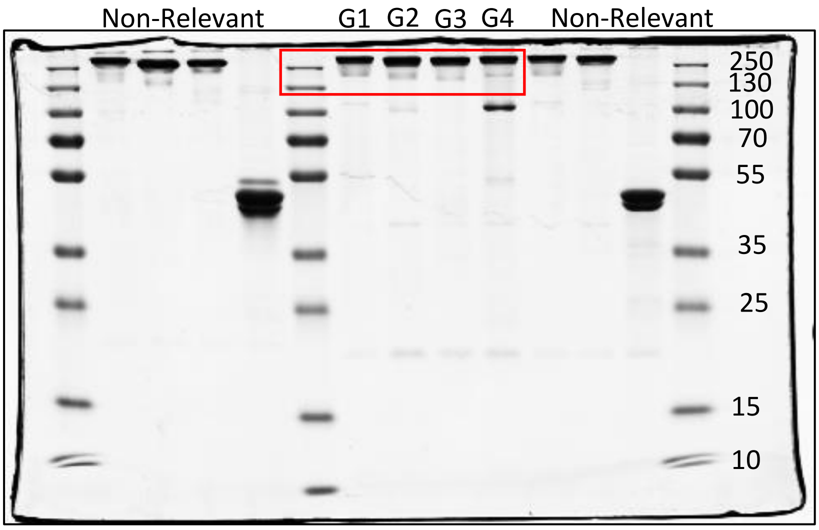

Non-Reducing Gel

Red boxes indicate the regions shown in the main figures. Other bands are not relevant to this paper.

Supplementary Figure 7

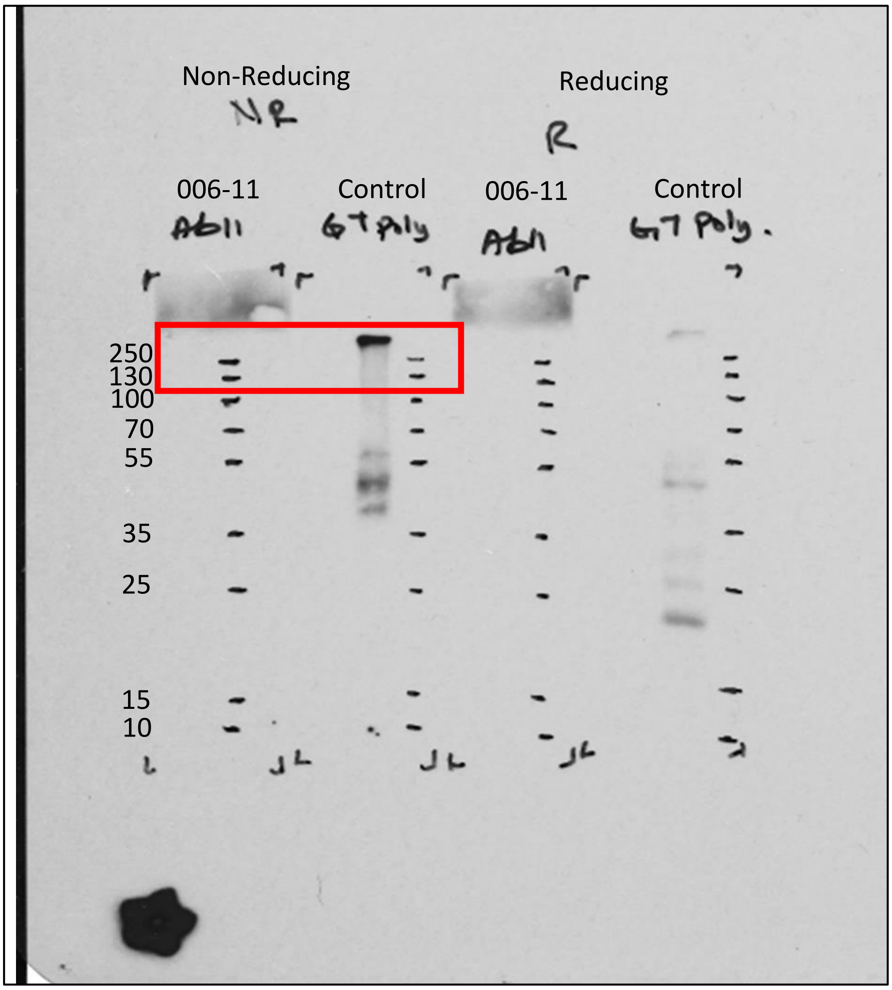

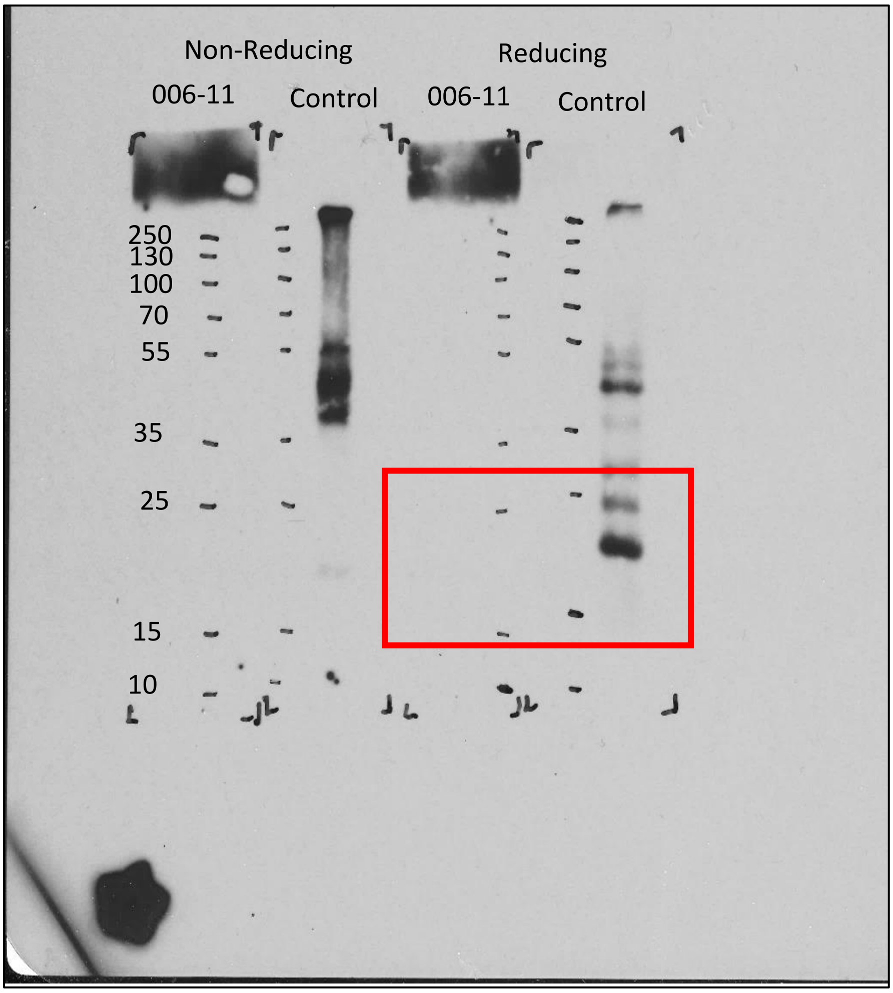

Red boxes indicate the regions shown in the main figures. Same Film was exposed for 15 seconds longer to make the reducing positive control bands more prominent (Bottom image).

Supplementary – Table 1

Table 1: Amino acid sequence of HBsAg (Genotypes)

Genotype	Amino acid sequence
A (adw2)	MENITSGFLGPLLVLQAGFFLLTRILTIPQSLDSWWTSLNFLGGSPVCLGQNS QSPTSNHSPTSCPPICPGYRWMCLRRFIIFLFILLLCLIFLLVLLDYQGMLPVCP LIPGSTTTSTGPCKTCTTPAQGNSMFPSCCCTKPTDGNCTCIPIPSSWAFAK YLWEWASVRFSWLSLLVPFVQWFVGLSPTVWLSAIWMMWYWGPSLYSIVS PFIPLLPIFFCLWVYI
B(adw)	MENIASGLLGPLLVLQAGFFLLTKILTIPQSLDSWWTSLNFLGGTPVCLGQNS QSQISSHSPTCCPPICPGYRWMCLRRFIIFLCILLLCLIFLLVLLDYQGMLPVCP LIPGSSTTSTGPCKTCTTPAQGTSMFPSCCCTKPTDGNCTCIPIPSSWAFAKY LWEWTSVRFSWLSLLVPFVQWFVGLSPTVWLSVIWMMWFWGPSLYNILSP FMPLLPIFFCLWVYI
C (adr)	MENTASGFLGPLLVLQAGFFLLTRILTIPQSLDSWWTSLNFLGGAPTCPGQN SQSPTSNHSPTSCPPICPGYRWMCLRRFIIFLFILLLCLIFLLVLLDYHGMLPVC PLLPGTSTTSTGPCKTCTIPAQGTSMFPSCCCTKPSDGNCTCIPIPSSWAFAR FLWEWASVRFSWLSLLVPFVQWFVGLSPTVWLSVIWMMWYWGPSLYNILS PFLPLLPIFFCLWVYI
D (ayw)	MENITSGFLGPLLVLQAGFFLLTRILTIPQSLDSWWTSLNFLGGTTVCLGQNS QSPTSNHSPTSCPPTCPGYRWMCLRRFIIFLFILLLCLIFLLVLLDYQGMLPVC PLIPGSSTTSTGPCRTCMTTAQGTSMYPSCCCTKPSDGNCTCIPIPSSWAFG KFLWEWASARFSWLSLLVPFVQWFVGLSPTVWLSVIWMMWYWGPSLYSIL SPFLPLLPIFFCLWVYI

Supplementary – Table 2

Table 2: List of antibodies used for cell sorting

Marker	Fluorochrome	Manufacturer	Clone	Catalog number
CD19	BV510	Biolegend	SJ25C1	363020
CD3	APC-Cy7	Biolegend	OKT3	317342
CD14	APC-Cy7	Biolegend	HCD14	325622
CD27	BV650	Biolegend	0323	302828
CD38	PE-Cy7	Biolegend	HB-7	356608
IgG	PerCP-Cy5.5	BD Biosciences	G18-145	624060
IgM	BUV496	BD Biosciences	UCHB1	750366
