# Supplementary material for: Defining the specificity and function of a human neutralizing antibody for Hepatitis B virus
Source: NPJ Vaccines. 2022 Oct 21;7:121. doi: 10.1038/s41541-022-00516-7 (PMC9586962; doi:10.1038/s41541-022-00516-7)
Supplement: Supplementary file 2 — REPORTING SUMMARY [file 41541_2022_516_MOESM2_ESM.pdf]

nature portfolio

Corresponding author(s): Dr Paul A. Macary

Last updated by author(s): 22/05/2022

Reporting Summary

Nature Portfolio wishes to improve the reproducibility of the work that we publish. This form provides structure for consistency and transparency in reporting. For further information on Nature Portfolio policies, see our Editorial Policies and the Editorial Policy Checklist.

Statistics

For all statistical analyses, confirm that the following items are present in the figure legend, table legend, main text, or Methods section.

n/a	Confirmed	
☐	☒	The exact sample size (*n*) for each experimental group/condition, given as a discrete number and unit of measurement
☐	☒	A statement on whether measurements were taken from distinct samples or whether the same sample was measured repeatedly
☐	☒	The statistical test(s) used AND whether they are one- or two-sided<br>*Only common tests should be described solely by name; describe more complex techniques in the Methods section.*
☒	☐	A description of all covariates tested
☒	☐	A description of any assumptions or corrections, such as tests of normality and adjustment for multiple comparisons
☐	☒	A full description of the statistical parameters including central tendency (e.g. means) or other basic estimates (e.g. regression coefficient) AND variation (e.g. standard deviation) or associated estimates of uncertainty (e.g. confidence intervals)
☐	☒	For null hypothesis testing, the test statistic (e.g. *F*, *t*, *r*) with confidence intervals, effect sizes, degrees of freedom and *P* value noted<br>*Give P values as exact values whenever suitable.*
☒	☐	For Bayesian analysis, information on the choice of priors and Markov chain Monte Carlo settings
☒	☐	For hierarchical and complex designs, identification of the appropriate level for tests and full reporting of outcomes
☒	☐	Estimates of effect sizes (e.g. Cohen's *d*, Pearson's *r*), indicating how they were calculated

Our web collection on statistics for biologists contains articles on many of the points above.

Software and code

Policy information about availability of computer code

Data collection	Attune NxT Flow Cytometer software<br>BD FACDiva Software
Data analysis	Graphpad Prism 6.0<br>Flowjo Software

For manuscripts utilizing custom algorithms or software that are central to the research but not yet described in published literature, software must be made available to editors and reviewers. We strongly encourage code deposition in a community repository (e.g. GitHub). See the Nature Portfolio guidelines for submitting code & software for further information.

Data

Policy information about availability of data

All manuscripts must include a data availability statement. This statement should provide the following information, where applicable:

- Accession codes, unique identifiers, or web links for publicly available datasets
- A description of any restrictions on data availability
- For clinical datasets or third party data, please ensure that the statement adheres to our policy

The datasets generated during and/or analysed during the current study are available from the corresponding author on reasonable request.

Field-specific reporting

Please select the one below that is the best fit for your research. If you are not sure, read the appropriate sections before making your selection.

☒ Life sciences ☐ Behavioural & social sciences ☐ Ecological, evolutionary & environmental sciences

For a reference copy of the document with all sections, see nature.com/documents/nr-reporting-summary-flat.pdf

Life sciences study design

All studies must disclose on these points even when the disclosure is negative.

Sample size	Sample size for mice experiments were determined based on previous experience with dosing humanized mice with therapeutic modalities. I
Data exclusions	1j - One dataset was left out due to 3 wells showing reduced readings for the top antibody concentration possibly due to pipetting error of flow antibodies into the wells during processing.
Replication	All experiments were repeated at least 3 times. The respective N numbers are indicated in the figures/methods.
Randomization	Once humanization was confirmed by hALB measurement, mice were randomly assigned to groups.
Blinding	Investigator was not blinded.

Reporting for specific materials, systems and methods

We require information from authors about some types of materials, experimental systems and methods used in many studies. Here, indicate whether each material, system or method listed is relevant to your study. If you are not sure if a list item applies to your research, read the appropriate section before selecting a response.

Materials & experimental systems

n/a	Involved in the study	
☐	☒	Antibodies
☐	☒	Eukaryotic cell lines
☒	☐	Palaeontology and archaeology
☐	☒	Animals and other organisms
☐	☒	Human research participants
☒	☐	Clinical data
☒	☐	Dual use research of concern

Methods

n/a	Involved in the study	
☒	☐	ChIP-seq
☐	☒	Flow cytometry
☒	☐	MRI-based neuroimaging

Antibodies

Antibodies used	Thermo Fisher Scientific, cat. #MA1-7606<br>Invitrogen cat. #A21235<br>Fitzgerald Cat. 10-H08A<br>Thermo Fisher Scientific, cat. #31413<br>Biolegend, Cat 363020<br>Biolegend Cat 317342<br>Biolegend Cat 325622<br>Biolegend Cat 302828<br>Biolegend Cat 356608
Validation	Thermo Fisher Scientific, cat. #MA1-7606 has been successfully used in ELISA, flow cytometry, immunoprecipitation and immunohistochemistry procedures.<br>Fitzgerald Cat. 10-H08A - Hepatitis B Virus preS2 antibody was raised in mouse using hepatitis B virus as the immunogen. Validation is also seen in Supplementary data Figure 1b.<br>Biolegend antibodies - Refer to company website for validation.

Eukaryotic cell lines

Policy information about cell lines

Cell line source(s)	HepAD38 cells - Cells obtained from collaborators lab<br>HepG2-hNTCP - Cells obtained from collaborators lab

Authentication	No authentication was carried out
Mycoplasma contamination	Cells lines were not tested for Mycoplasma contamination
Commonly misidentified lines (See ICLAC register)	*Name any commonly misidentified cell lines used in the study and provide a rationale for their use.*

Animals and other organisms

Policy information about studies involving animals; ARRIVE guidelines recommended for reporting animal research

Laboratory animals	FRG KO mouse strain was used in this study. Randomized male and female mice aged ~5-6 months old displaying ~70% repopulation of human hepatocytes were utilized for animal experiments. More detailed information can be found in the materials and methods section under 'Generation of human liver chimeric mice'.
Wild animals	*Provide details on animals observed in or captured in the field; report species, sex and age where possible. Describe how animals were caught and transported and what happened to captive animals after the study (if killed, explain why and describe method; if released, say where and when) OR state that the study did not involve wild animals.*
Field-collected samples	*For laboratory work with field-collected samples, describe all relevant parameters such as housing, maintenance, temperature, photoperiod and end-of-experiment protocol OR state that the study did not involve samples collected from the field.*
Ethics oversight	The International Animal Care and Use Committee (IACUC), A*STAR specifically approved this study under the protocol number #181367. All animal experimental procedures were conducted in accordance to IACUC's guidelines.

Note that full information on the approval of the study protocol must also be provided in the manuscript.

Human research participants

Policy information about studies involving human research participants

Population characteristics	Human peripheral blood was obtained after informed consent from an acute-recovered HBV patient (DSRB 2015/00354, Hepatitis B virus eradication and loss (HEAL) cohort study).
Recruitment	It was part of a cohort study run by National University Hospital Singapore.
Ethics oversight	Study protocols are approved by the National University of Singapore Institutional Review Board.

Note that full information on the approval of the study protocol must also be provided in the manuscript.

Flow Cytometry

Plots

Confirm that:

☒ The axis labels state the marker and fluorochrome used (e.g. CD4-FITC).

☒ The axis scales are clearly visible. Include numbers along axes only for bottom left plot of group (a 'group' is an analysis of identical markers).

☐ All plots are contour plots with outliers or pseudocolor plots.

☒ A numerical value for number of cells or percentage (with statistics) is provided.

Methodology

Sample preparation	Cells were trypsinized from the plate at 7 days post infection and fixed. Cell were stained in FACS buffers.
Instrument	Attune NxT flow cytometer<br>BD FACSAria III cell sorter
Software	Analysis - FlowJo software
Cell population abundance	Post sort cell analysis was confirmed by running a small sample of the sorted cells again by flow cytometry.
Gating strategy	Please refer to figures

☒ Tick this box to confirm that a figure exemplifying the gating strategy is provided in the Supplementary Information.
